# Client Experiences in a Mobile-Phone Counseling Intervention for Enhancing Access to Prevention of Mother To-Child Transmission (PMTCT) Services in Kenya

**DOI:** 10.3389/fgwh.2022.785194

**Published:** 2022-06-03

**Authors:** Jerry Okoth Okal, Avina Sarna, Daniel Lango, James Matheka, Danmark Owuor, Eunice Auma Kinywa, Sam Kalibala

**Affiliations:** ^1^Population Council, Nairobi, Kenya; ^2^Population Council, New Delhi, India; ^3^Independent Consultant, Nairobi, Kenya; ^4^Ministry of Health, Kisumu, Kenya; ^5^Population Council, Washington, DC, United States

**Keywords:** cell-phone, PMTCT, HIV, women, intervention

## Abstract

**Background:**

The prevention of mother-to-child transmission (PMTCT) is considered one of the most successful HIV prevention strategies in detecting and reducing HIV acquisition *in utero* or at birth. It is anticipated that with the increasing growth of digital technologies mobile phones can be utilized to enhance PMTCT services by improving provider-client interactions, expanding access to counseling services, and assisting in counteracting social and structural barriers to uptake of PMTCT services. Understanding the subjective experiences of women accessing PMTCT services in different settings has the potential to inform the development and promotion of such methods. This paper explores the perspectives of HIV-positive pregnant women attending maternal and neonatal clinic services in Kisumu, Kenya.

**Methods:**

Data are reported from in-depth interviews with women, following a longitudinal study investigating the impact of a structured, counselor-delivered, mobile phone counseling intervention to promote retention in care and adherence to ARV prophylaxis/treatment, for HIV-positive pregnant women. Thematic content analysis was conducted.

**Results:**

Discussions indicated that mobile-phone counseling provided useful health-related information, enhanced agency, and assisted mothers access critical PMTCT services across the cascade of care. Similarly, mobile-phone counseling offered personalized one-to-one contact with trained health providers including facilitating discussion of personal issues that likely affect access to services. Findings also identified barriers to the uptake of services, including a lack of partner support, poor health, poverty, facility-related factors, and provider attitudes.

**Discussion:**

Overall, findings show that mobile-phone counseling is feasible, acceptable, and can enhance access to PMTCT services by overcoming some of the individual and facility-level barriers. Although mobile-phone counseling has not been routinized in most health facilities, future work is needed to assess whether mobile-phone counseling can be scaled-up to aid in the effective use of HIV and PMTCT services, as well as improving other related outcomes for mother and child dyad.

## Introduction

Mother to child transmission (MTCT) continue to undermine the progress made in confronting the HIV pandemic in highly burdened sub-Saharan Africa countries. Evidence from some SSA countries demonstrates that from the time of registration at the antenatal clinic and delivery, the proportion of loss to follow-up is still high (1, 2). Kenya has made significant steps to expand the coverage of PMTCT of services, evidenced by the decline in infant HIV transmission from 16% in 2012 to 6.9% in 2015 (3) However, Kenya has yet to achieve the global target of reducing perinatal transmission to 5% (4). One of the main challenge for the PMTCT program is that some mother-baby pairs fail to attain the desired treatment outcomes because they rarely follow through the full continuum of services recommended during antenatal and postnatal visits (5). In some instances, it may be difficult for women to initiate highly active antiretroviral treatment (HAART) or be retained in PMTCT care as they are constrained by individual, structural, and health facility-related barriers (6–10).

Counseling is an integral component of patient management in HIV care and treatment programs; however, the focus of most counseling sessions is largely geared toward preparing patients for HAART and supporting them through the first weeks of treatment initiation. After that patients are often left on their own to focus on treatment which likely causes confusion resulting in health and social challenges that may affect treatment outcomes. To be responsive, counseling can be tailored to be patient-centered, interactive, and cover a specific period beyond the treatment initiation phase. With the upsurge in the use of digital environments and related information and communication technologies, PMTCT programs can use mobile phones to offer targeted counseling to achieve positive treatment outcomes. However, there is little known about user perspectives and the utility of a mobile phone delivered counseling model aimed at improving treatment outcomes through the cascade of PMTCT services.

The use of mobile communication in enhancing health outcomes has shown success in improving HIV testing and antiretroviral therapy adherence in different settings (11–13). The high potential of wireless communication technologies in health programs has also been identified by the United Nations Joint Programme on HIV/AIDS (UNAIDS) and WHO (13). The use of digital technology in the HIV cascade and specifically in PMTCT care has proven to enhance access to services, retention, and adherence to HAART. However, some studies have had mixed results due to a combination of factors including access, saturation, type of technology deployed and the content among others (44–48). In Kenya, a two-way mobile phone short message service has been used successfully to promote ART adherence (13) and weekly one-way communication from provider to client used to improve adherence outcomes (14). In Sub Saharan Africa, a wide range of PMTCT services could be enhanced by providing targeted mobile-phone counseling through the cascade of care.

We evaluated the effectiveness of a structured, counselor-delivered, mobile phone counseling intervention to promote retention in care, adherence to ARV prophylaxis/treatment, and uptake of HIV testing of infants at 6 weeks among HIV-positive pregnant women compared to standard care in Kenya (15). In this paper we present findings from 27 in-depth interviews (IDIs) that were conducted with sub-groups of women who took part in the main study, to better understand their experience and perspectives regarding PMTCT services, retention in care, and the mobile-phone counseling intervention. The population-level uptake of mobile phone counseling is likely context-specific, as trade-offs between positive and negative attributes are shaped by individual, social-cultural, structural, and health facility factors. More so, user attributes such as education, age, marital status and socio-economic status can enhance access to and use of mobile devices. The subjective experiences of women accessing PMTCT services in different settings have the potential to inform the development and promotion of such methods. This paper explores the perspectives of HIV-positive pregnant women who participated in a randomized cohort study to provide additional qualitative insights that could not be obtained through quantitative surveys.

## Methods

The qualitative interviews were conducted with women who participated in the Healthy Mother Healthy Baby project (HMHB), a two-arm randomized controlled study investigating the effect of structured phone counseling (the intervention) to promote retention in care until 15 weeks postpartum and adherence to ARV prophylaxis/treatment for HIV-positive pregnant women compared to standard of care (treatment as usual) (15). A total of 404 HIV-positive pregnant women attending antenatal care (ANC) at 14 PMTCT study sites in and around Kisumu were recruited into the study. Participants recruited into the study were randomly assigned to the intervention (n = 207) and control arms (n = 197). All study participants received a minimum standard of care services including routine ANC, delivery, and postpartum care, PMTCT, and HIV care at the mother and child health (MCH) clinic (16).

### The Health Mother Healthy Baby (HMHB) Intervention

The intervention of the study was mobile phone counseling delivered to women enrolled in the study through PMTCT services at 14 health facilities enabling the participants to have frequent, personalized one-to-one contact with a trained counselor over and above contact during clinic visits. The counselors were based at an off-site location, were introduced to the clients by clinic nurses and study staff over the phone and provided phone counseling without meeting the clients. Besides counselors were trained on the national ART adherence counseling manual, used by nurses at the clinic, and on strategies to approach HIV-positive mothers on the phone, confidentiality, targeted-need-based counseling, and identifying mothers at risk of defaulting treatment. Each counselor served an average of 40 clients throughout the study period. Counselors encouraged women to attend all antenatal visits, to deliver at the institution, and reminded them of their scheduled appointments and to bring back their newborns for HIV testing at 6 weeks. The counseling plan entailed: two phone calls in the first week of enrollment in ANC followed by one weekly phone call until delivery (maximum of 24 calls depending upon the weeks of pregnancy). Post-delivery counseling consisted of two phone calls in week one, and 1 weekly phone call until 15 weeks post-partum (maximum of 16 calls). Post-delivery intervention timeline of 15 weeks post-partum was used because the main study was assessing retention at 6 and 14 weeks postpartum. Phone counselors maintained a confidential log of all calls made and issues discussed (date, duration, key issues discussed and action plans). Participants were free to call the counselor (client-initiated calls) at any time and request the counselor to call back about any problems they faced. All phone calls were made free of charge to the participant. It is estimated that each call lasted between 5 and 20 min. While phone counselors were not based at clinics, they worked closely with the nurses at the MCH clinics, through phone contact, to coordinate issues regarding clients as necessary. During counseling, participants asked questions/raised concerns related to ART initiation, adherence, and PMTCT (e.g., side effects, disclosure issues, breastfeeding) that manifested in between clinic visits and counselors assisted in exploring these challenges and possible solutions. The project engaged five trained counselors (with diverse backgrounds inpatient care such as HIV testing and counseling) and trained them to use the intervention's counseling operational guide (with a standard set of questions and checklists) to counsel clients. The IDIs explored experiences accessing PMTCT services and the utility of mobile-phone counseling in enhancing access to PMTCT services. Findings from the main study [*n* = 404 participants, (*n* = 207, intervention and *n* = 197, control arm)] showed the counseling intervention (hazard ratio [HR] = 0.29; 95% confidence interval [CI] = 0.12, 0.69) and positive health perceptions (HR=0.99; 95% CI=0.98, 1.00) were associated with lower hazards of being lost to follow-up (17).

### Data Collection and Analyses

IDIs were conducted with a subset of 27 purposively selected participants representing different categories as listed in Table 1 (We aimed to interview 2–4 clients per category, but in some instances this target was not achieved. Some women were unavailable for an extended duration while others declined interviews). Women representing different categories were selected owing to their different contexts to obtain varied perspectives and experiences. The participants were mainly drawn from health facilities within Kisumu County and its environs. IDI's were conducted by experienced qualitative interviewers using a semi-structured interview guide to facilitate the discussion. The guide explored clients' experiences with ANC, clinic attendance, delivery, infant feeding, immunization, and adherence. Additionally, participants were asked to discuss their experience with phone counseling and the HMHB project. IDIs were conducted in Kiswahili and Luo languages, audio-recorded, transcribed, and translated into English. Thematic content analysis was used for the subjective interpretation of the content of text data through the systematic classification process of identifying themes, sub-themes, or patterns (18). Themes identifying key factors influencing the routine use of PMTCT services including facilitators and challenges, and how mobile-phone counseling supported clients and enhanced agency were identified by research staff (JO, DL, AS). Identification of themes was an iterative process whereby codes and themes were redefined or merged based on emerging patterns in the data (19).

**Table 1:** Participants IDI categories.

	**Category**	**No of IDIs**
1	Attended ANC regularly but chose to deliver at home	2
2	Attended ANC regularly and delivered in the Health Facility	1
3	Attended ANC irregularly and delivered at home	1
4	Attended ANC irregularly but chose to deliver at the Health facility	3
5	Came for the PNC (Post Natal Care) at 6 weeks and got the infant tested	4
6	Delayed the PNC and/or did not get the infant tested	3
7	Registered at ANC (14–20 weeks) (early registration)	2
8	Registered at the ANC (28–32 weeks) (late registration)	5
9	Have other children and have accessed PMTCT services	3
10	Women who had a stillbirth	1
11	Women who had late ANC attendance	1
12	Women who were lost to follow-up but found later	1
	**Total**	27

### Ethical Approval

This protocol was approved by the Population Council's Institutional Review Board, the National Commission for Science, Technology, and Innovation (NACOSTI) and the Kenyatta National Hospital/University of Nairobi Ethics & Research Committee. Written informed consent was obtained from all participants.

## Results

The Content Analysis Highlighted Several Themes Concerning Women's Subjective Experiences With Accessing PMTCT Services Including the Challenges and Strategies Used in Accessing Care in Health Facilities in Kisumu County. Many of These Themes Underscore how Mobile-Phone Counseling Enhanced Women's Agency to Utilize PMTCT Services and Take Appropriate Health Actions for Themselves and Their Children. Similarly, Phone Counseling Intervention Offered Targeted Health Advice on Adherence to ARVs/Nevirapine, Regular Attendance at Clinic Visits, Institutional Delivery, Child Health, Exclusive Breastfeeding, and Compliance With Child Immunization Requirements in Addition to Routine Care Offered in PMTCT Services.

## Adherence to ARVs

In general, ARVs require a high level of adherence to minimize treatment failure and viral resistance. To comply with this requirement, most women indicated that they achieved adherence by utilizing multiple strategies. For example, to minimize the risk of missing doses of ARVs at designated times, most participants asserted that social support from people close to them such as family members and friends, as well as the use of electronic devices such as alarm clocks and phones helped them take medications on time. Participants who had disclosed their status to their partner/spouse benefited from their support, as described below.

“*When I do not have medication or my medication is almost over he (husband) tells me that my medication is almost done and will only cover me for a specific period… I was open with my husband and there was no difficulty, he sat down with me and also told me that such kinds of things happen and I should not think about it so I did not think about it”*

*062015 (Married female, 25 years old, three children; Delayed the PNC and/or did not get the infant tested)*

…* I did set an alarm on my phone at 7 am when it rings I take the drugs*

*(Unmarried female, 27 years old, two children:* Registered at the ANC (28-32 weeks) (late registration).

Besides utilizing other strategies to adhere to treatment, it was evident from the narratives that, mobile-phone counselors interacted with participants and made concerted and conscious efforts to constantly remind them to take their medications. Nearly all participants affirmed that phone counselors regularly contacted them over the phone and advised them to take ARVs and give Nevirapine to their HIV-exposed infants, as cited below.

“*The counselor is proceeding on with me well, even sometimes she calls me to ask how I am faring on and how the baby is faring on if I am giving the baby medication well if I am taking my medication well. Even when the baby is sick she tells me just to have hope that the baby will be well”. (Married female, 31-year-old, lived in Kisumu 3 years:* Came for the PNC at 6 weeks and got the infant tested*)*

“*Yes, if you are late if you are supposed maybe to take it (drug refill) tomorrow or you were supposed to go today and you have not gone they call you (mobile-phone counselors) on the phone”*.

*(Single female, 26-year-old:* Delayed the PNC and/or did not get the infant tested)

Consistent use of ARVs was often linked to better health outcomes including increased appetite, ability to undertake daily chores, and improved social relations. Consequently, motivation for sustained use of ARVs was often associated with being alive, productive, confident, and healthy as explained below.

“*…(with ART) I do not get sick, I eat well and have an appetite, I work normally and I do not feel tired*

*(Married female, 30-year-old-:* Attended ANC regularly but chose to deliver at home “*Initially I used to be sickly and could not perform some chores, but I am happy I am strong and can do all chores effectively. I am strong and I have gained weight compared to the way I was thin”*. Have other children and have accessed PMTCT services

*(Married female, 31- years- old* Attended ANC regularly but chose to deliver at home*)*

Disclosing their status with their spouses also helped women feel stronger and more confident about themselves, as this 31-year-old married participants reports:

“*I used to fall sick and my husband could ask me why, I knew of my status but he did not but after he knew about it, at least I became more confident… (Married female, 31-year-old:* Registered at ANC (14–20 weeks) (early registration)

However, medication adherence and compliance with other health requirements was a challenge for some women, especially those in a union or long-term partnerships who had not disclosed their HIV status to their partners. Non-disclosure of status often resulted in a lack of support from the partner and was closely linked with improper or erratic use of ARVs because of fear (including fear of receiving calls from the phone counselor) and frequent quarrels. The quotes below describe the experiences of women who had difficulties disclosing their HIV status to their partners:

“*Because initially, I thought to myself what if the person I live with comes across this medication, as in I was scared of quarreling that maybe I would have to quarrel, even as I spoke to my advisor (mobile-phone counselor) from the town I preferred telling her to call me at a time she thought the person I live with was not around”. (Cohabiting female, 19-year-old:* Have other children and have accessed PMTCT services)

*Some participants who did not have any support reported missed doses: “There are times I… sometimes I forget in the morning and when I forget I take all of them the four at night.” (Married female, 35-year-old: Have other children and have accessed PMTCT services)*

## Use of Nevirapine by Infants

Regardless of where women delivered their children (e.g., at home with a traditional birth attendant or health facility), most women emphasized that they administered Nevirapine to their children as instructed by the health professionals and phone counselors. Thus, most participants affirmed that they administered Nevirapine immediately after birth or within the shortest time possible after delivery. To a large extent though, compliance with Nevirapine was directly linked to women's knowledge and awareness of the importance of Nevirapine to the child's health. For example, the quote below portrays an account where a woman with an imminent caesarian section reminded providers to give Nevirapine to the unborn/newly born child.

*Interviewer: Okay, have you ever missed giving it (Nevirapine) to the baby?Respondent: Only when the baby was born because I had been taken for an operation (cesarean section), so when the baby was at the nursery I reminded them (nurses) when…no I had told them before going to the theatre but they forgot, the medication was started on the second day (Married female, 26-year-old:* Attended ANC irregularly but chose to deliver at the Health facility*)*

Although knowledge on Nevirapine' was nearly universal, it is apparent from the narratives that frequent interactions with mobile-phone counselors enhanced women's knowledge of its benefits. On several occasions, counselors educated women and emphasized the importance of Nevirapine by constantly reminding women to give this medication to their newborn children.

## Clinic Attendance

Most women felt that the *advice* and *reminders* provided by mobile-phone counselors were useful and ensured that they attended the ANC clinic regularly, collect ART medications and access critical health services promptly.

“*I was always told (by mobile-phone Counsellor) to attend (clinic) so that I can always get my drugs to keep me strong. Anytime I came they could measure our weights and advise us how to take care of the baby”. (Married female, 24 years old: Attended ANC irregularly and delivered at home)*

“…She (mobile-phone counselor) tells me not to be late for the clinic… (Inaudible) I should see the doctor and everything else”. (Married female, 24 years old with 2 children: Attended ANC irregularly and delivered at home)

Additionally, participants mentioned that constant reminders from phone counselors before or up until the very day of the clinic appointment helped them plan well ahead of time thereby saving time and being adequately prepared for each clinic visit.

“*They also tell me when next I should be visiting the clinic. They even remind me on the very morning of the clinic day……..It has been helpful, as I cannot waste my time to go to the clinic when it is not the day I am supposed to visit. This also helps me note the dates and prepare adequately” (Married female, 35 years old with three children:* Attended ANC regularly but chose to deliver at home*)*

Although phone counselors talked regularly with their clients and reminded them to make regular clinic visits, some women continued to experience difficulties fulfilling this requirement for several reasons; foremost among these was the lack of spousal support, distance to the health facility, the cost for transportation, as well as provider attitudes, as illustrated below

*Interviewer: Yes, and regarding the clinic how does your husband help you regarding the clinic?Respondent: Nothing, when I tell him that I am going to the clinic he tells me to just go(Married female, 22- years- old:* Attended ANC regularly and delivered in the Health Facility*)*“*(the first time I went to the clinic) I went after five months…You know where I am going is far, so I only go when I have money, so it forced me to go after five months” (Married female, 30- years- old: 041087* Attended ANC regularly but chose to deliver at home “*…They (the providers) were not happy with me and they even quarreled with me. The following month I did not go and that is when I delivered…at home*.

*(Married female, 24- years- old with 2 children)*

Despite the efforts made by counselors to underline the importance of regular clinic attendance, a few women did not take clinic attendance seriously. This was based on their previous successful birth experiences for which they did not attend scheduled ANC visits and did not experience complications during birth. Therefore, in some cases, women with prior birth experiences felt that clinic attendance was not necessary before delivery.

*Interviewer: Why were you late in coming to the clinic? Why did you not come early?Respondent: I was just used to it, even my first pregnancy I never went to the clinic, I was like I will just go to give birth…. because I knew that there was no problem it is just that I was used to it. (Unmarried female, 27- years- old:* Registered at the ANC (28-32 weeks) (late registration)

## Health Facility Delivery

The phone counselors were trained to emphasize the importance of health facility delivery and advise women about the closest health facilities in the community. The guidance for institutional delivery was client-centered, considered client preference and proximity of facilities to the client's homes. Overall, during counselors-clients interactions, personal reasons emerged as a key influencer in women's decision to deliver at a specific health facility. Key among these reasons was experience (s) with a previous pregnancy, health facility characteristics, peer influence, and spousal support. From most accounts, it was evident that past pregnancy experience (either good or bad experience) had a bearing on the choice of point of delivery. For example, women who had previously experienced a birth complication almost always elected to deliver at the health facility to avoid future complications during delivery.

“*I delivered at [Facilit X] since I had the previous complication and this delivery could also be difficult. This made me choose it, and I was taken for a C-Section”(Married female 23- years- old, unmarried* Have other children and have accessed PMTCT services)

Similarly, as narrated in the quote below, the choice of a point of delivery for some women occurred due to the influence of people close to them, such as partners or peers, who often advised them to deliver at a facility based on their reflection of the woman's past pregnancy experiences. In the quote below, one woman recalls receiving multiple sources of advice to deliver at a health facility.

“*It's my husband who told me that as I had an operation previously just to go and give birth at the hospital, other people also told me, the counselor also told me just to give birth at the hospital because of the baby and because of the medications I should not give birth at home”*.*(Married female, 27- years -old with three children:* Registered at the ANC (28-32 weeks) (late registration)

In other instances, phone counselors influenced women's women's decision to deliver at a health facility based on a careful and detailed assessment of risk during their interactions with the clients.

*I was told and advised by (mobile-phone counselor name) to deliver at the hospital. I also thought it wise to do so since delivering at home could be risky to me. (Married female, 24 years old:* Have other children and have accessed PMTCT services)

Perceived provider's attitudes also appear to have had a big bearing on women's' decisions to deliver at a health facility. Often, women selected health facilities where they felt welcome and were confident that staff would be responsive and handle them well during delivery. Further, women selected facilities that they had previously heard about and perceived to offer superior quality of care and were clean and hygienic.

“*The reason why I saw it [the facility of choice] as being good is that when I was pregnant I felt that those guys [from another facility] did not take my case seriously, and if I went to give birth there maybe they would have been like “no this is not due this pregnancy is not yet due” I would even have been told to go back home I just thought I should go to [facility of choice](Married female, 26- years- old: Came for the PNC at 6 weeks and got the infant tested)*

“*We were told to single out where we would like to deliver. I chose _______ because I liked it…..I was told by other women who have delivered there before that it is a clean place (Married female, 26 years old* Attended ANC regularly and delivered in the Health Facility*)*

“*The Facility_________. When I went to visit my friend, I found it to be clean, the nurses were so professional and I had to go for it.”(Married female, 26 years old* Attended ANC irregularly but chose to deliver at the Health facility*)*

Even though most women were aware of the importance of institutional delivery, our data indicate that there were two main drawbacks to delivering at a health facility. Notably, these were abrupt/unexpected onset of labor and inaccurate projection of the delivery date. While some women planned and desired to give birth at a health facility, they were unable to because of the abrupt onset of labor that practically scuttled plans to reach the health facility on time. The following quotes illustrate how abrupt and unexpected onset of labor hindered access to health facilities.

“*I did not know that my water was going to break I did not see any signs…So at one in the morning, I started having stomach upsets and after three times I suddenly saw that the baby was already out. (Married female, 23- years- old:* Attended ANC irregularly and delivered at home*I was alone in the house and my water broke at three o'clock in the morning, I had never experienced anything so when it was at three o'clock in the morning, I felt a bit of stomach upset and I had the contact of a midwife when I called she told me to wait up to five o'clock in the morning…On reaching four o'clock in the morning, I gave birth alone”. (Married female, 25 years old:* Attended ANC regularly but chose to deliver at home*)*

Besides, some women failed to deliver at a health facility because of inaccurate projection of their expected date of delivery by providers or themselves.

“*I went to the hospital in the morning (of the day of delivery) and they told me that the time to give birth had not reached that I had approximately two weeks… When it was at night at around midnight my stomach started aching so you know we are far from the road so to come from there to reach here was hard so I gave birth just at home”. (Married female, 24- years- old Attended ANC regularly but chose to deliver at home*

“*My labor pains are instant, they don't last long, and as such, I deliver immediately. We had a funeral nearby, and as we went to pick the body, I had known of delivering in May and not April. I felt fierce stomach pain. So by the time I felt of going to General (hospital), the baby was already getting out, that is when I was taken to the midwife” (Married female, 24- years- old with 2 children)*

## Breastfeeding

In general, most participants were equipped with information on infant feeding guidelines owing to exposure to prevention messages and regular interaction with providers. However, despite this, the narratives illustrate how mobile-phone counselors reinforced messages on exclusive breastfeeding.

*She (Counsellor) told me to breastfeed until 6 months are over. I should not even give the baby water since the milk had everything the baby needed. (Married female, 26 years old)* *I was told to breastfeed until 6 months. Thereafter I was to take it (baby) back for testing and checkup to introduce other meals. The nurses told me that breast milk was healthy in boosting the child immunity system for 6 months. Adequately (Married female, 31 years old with three children: Attended ANC regularly but chose to deliver at home)*

Despite the high level of engagement that counselors had with clients, some women failed to follow infant feeding guidelines owing to underlying health or structural issues. For example, some women were incapable of breastfeeding because they were unwell (e.g., painful breasts), the infants refused to breastfeed, or they were unable to produce enough breast milk due to poor nutrition and conflicting advice from providers. The following quotes portray two women who instead of following infant feeding guidelines opted instead for mixed feeding practices primarily due to the inability to produce milk and poor health.

“*(I started breastfeeding) Immediately after delivery, after 3 months there were some growths that developed that could even bleed at times. They told me to stop breastfeeding and give him food. And I stopped breastfeeding after 3 months”. (Married female, 36- years- old:* Registered at ANC (14–20 weeks) (early registration)

Another respondent reported,

“*And because I am sick (one painful breast), I cannot only breastfeed because the baby only suckles from one breast the other breast is not producing breast milk, so I also give the baby porridge…The baby cannot get infected because the baby takes medication and I am also taking my medication, I think there is no way…” (Married female, 25 years old:* Delayed the PNC and/or did not get the infant tested*)*

For some women, the inability to produce enough breastmilk was closely linked to lack of food and poor nutrition linked with underlying poverty. Therefore, regardless of women's knowledge and awareness of the importance of breastfeeding, those from poor households had limited options to practice exclusive breastfeeding.

*Just that sometimes when food was scarce, if I did not eat well I could see that the breast milk was not sufficient as the baby suckled after which the baby started crying…So sometimes, I called my mother, if she had money she sent it to me. (Unmarried female, 19 years old:* Delayed the PNC and/or did not get the infant tested.

## Child Immunization

Overall, most women reported that they had immunized their infants as per the national immunization schedule. However, women's accounts indicate that frequent mobile-phone interactions with the counselors enhanced their knowledge and helped them adhere to scheduled immunization dates.

“*Yes, the baby got all of them even all the injections the baby has finished(Married female, 24- years- old:* Came for the PNC at 6 weeks and got the infant tested*)*

“*Yes, I did as I was advised by (name of a counselor). The first one the baby received some drops in the mouth and I took her for the rest of immunization in the clinic without delay” (Married female, 23- years- old* Attended ANC regularly and delivered in the Health Facility*)*

## Acceptability of Mobile Phone Counseling

Almost all participants expressed satisfaction with the HMHB program which they said helped them improve their own and their children's health. The phone counseling also offered women an opportunity to openly discuss and share their problems with counselors who listened and helped them work through their problems and provide possible solutions; more importantly, women could reach out to the counselor (when needed) between the routine call schedule. These one-on-one interactions fostered a community of support for women which is normally rare or non-existent within the community or formal health settings. The following quote captures the personal benefits attributed to the HMHB project.

…* “I see That Since We Started…Since I Joined the Program I Feel It Has Been Beneficial Because When I Am Going Through Difficulties Sometimes We Share and Talk and After Sharing I Feel Better Because I Found Someone That I can Talk to who can Give Me Advice….Sometimes When I Am Sick I Tell Her, She Tells Me…She Has Been Advising Me Frequently…..Yes, She Even Tells Me to Go to the Hospital Whenever I get Health Complications and not to Keep Quiet and if I Am Going Through any Difficulties to Share With Her”. Delayed the PNC and/or Did not get the Infant Tested (Unmarried Female,19 Years old)*

Furthermore, women who participated in the program said that they were satisfied with the frequency and quality of communication they had with phone counselors. Although phone counselors were expected to follow a call schedule, however, from the onset women were informed that they were free to initiate calls to their designated counselor whenever they desired. This resulted in many women initiating calls to the counselors to discuss their health or social problems that they would have otherwise opted not to discuss with anybody.

“*….even when I am facing any difficulties I call the counselor and I ask her then she tells me what to do, so there is no problem that I can say I have seen”. 060711 Attended ANC irregularly and delivered at home (Married female 24 years old, with 2 children)*

Hence, over time, counselor-client bonds grew and were strengthened by frequent communication resulting in an open discussion about social and health issues and overall satisfaction with counseling services. Most clients said that they were happy with the mobile-phone interactions and described counselors in positive terms such as “good”, “kind” and “attentive”. The following quote illustrates how the bond grew over time.

“*… They always check on me by calling to find out how I feed the baby and if I am having any challenges with taking drugs and if I can afford meals. They even encourage me which makes me more confident and enthusiastic…”. (Married female, 31-year-old* Attended ANC regularly and delivered in the Health Facility)

## Discussion

Although PMTCT programs have recorded remarkable achievements over the years, it has been shown that a range of individual, social, and structural factors influence the uptake of and retention in PMTCT services, including improved communication and contact with the health system, health education, partner and peer support, and patient advocacy and assistance (20). This study explored the experiences of women receiving a one-on-one individually tailored, theory-based, counseling intervention to improve retention in care, adherence to treatment, and the uptake of HIV testing of infants at 6 weeks in Kisumu, Kenya. The HMHB counselor-delivered, mobile phone counseling intervention emphasized the importance of patients' perceptions about their illness and treatment and attendant cognitive, emotional, behavioral, and contextual factors that influence adherence behavior (21). Our findings show that client motivation/agency to access PMTCT services, disclosure concerns, knowledge, and awareness of the importance of ART for the mother and Nevirapine for the infant, support from partners and peers, providers attitudes, and structural factors such as poverty and distance to health facilities remain key factors influencing the uptake of PMTCT services. Yet, mobile-phone counseling intervention enhanced women's agency to access critical PMTCT services and was acceptable and well-received by clients. However, underlying structural factors such as poverty, distance from health facilities, and individual-level factors (stigma, cost of services, and knowledge) remain barriers. This together with underlying concerns around disclosure and partner support require considerable attention when implementing a similar project on a wider scale.

The mobile-phone counseling intervention empowered women to seek health services and deal with individual and structural barriers across the cascade of services. In particular, mobile-phone counseling accorded women a medium to openly discuss important health and social issues with unbiased, sympathetic, knowledgeable counselors at a time of their convenience which they would have otherwise struggled to get at the community or within formal health facility setting. The narratives indicate that phone conversations helped educate and advise women to take appropriate steps to address their health concerns, plan well ahead of time, and work through their schedules to honor clinic appointments. While a few women delivered at home because of abrupt onset of labor and inaccurate estimation of the due date, nearly all women said they knew the importance of delivering at a health facility to forestall a birth complication. They also reiterated the importance of administering the first dose of Nevirapine to the child immediately after birth. Thus, regardless of the point of delivery, most women administered Nevirapine to the child immediately after delivery, highlighting women's awareness of the risk of HIV transmission and knowledge of important health messages. As previously documented, most women adhered to the guidance regarding exclusive breast-feeding (for the first 6 months of the infant's life), however, some women could not comply due to ill-health, (e.g., painful breast, and poor health), the child's poor health (e.g., refusal to breastfeed due to underlying health problem) or due to underlying structural factors which inhibited access to proper nutrition (e.g., inability to produce enough breast milk due to poor nutrition rooted in pervasive poverty) (22, 23). Several factors have been found to affect exclusive breastfeeding, in developing countries; maternal age, education, employment, residency, cultural and religious practices, antenatal care practices, home delivery, anxiety, and sickness of the mother (24–27). However, evidence suggests the factors that influence exclusive breastfeeding practice are context-specific and might differ from one setting to the other, necessitating the need for setting specific data (26). Past birth experiences where women had normal births and healthy children despite incomplete ANC attendance and non-institutional deliveries, appeared to influence the uptake of ANC in the current pregnancy; however, the phone counseling intervention did address this with mixed outcomes/success. Interestingly though, nearly all women diligently followed the child immunization schedule and immunized their children per the laid-out schedule. In Kenya, the immunization coverage has been steadily rising from 57% in 2003 to 77% in 2007 (28).

Our study findings are in line with existing evidence on mobile phone interventions, which has increasingly shown that wireless communication can enhance uptake of health services for different population groups such as HIV positive individuals, pregnant women and the youth (29–31). The HMHB project offered a two-way communication line between women attending PMTCT services and counselors, including unstructured phone calls initiated by clients which provided women with personalized support whenever they needed it—as they did not have to visit the center to access similar support. Besides, discussion of hitherto private social problems might have helped women address other social problems they were struggling with. For example, other research findings highlight how disclosure of HIV status and lack of partner's support may inhibit access to PMTCT services (32). Similarly, the findings from this study can provide insights into the use of digital technology in the provision of health services. This is more evident during the Covid-19 pandemic where telemedicine/tele counseling proved to be useful in many low and middle income countries such as Kenya.

While the counselor-client encounters enhanced women's personalized support, some specific health improvements can be attributed to mobile-phone counseling: women were able to identify labor signs early, regularly visit the clinic for ANC and PNC, deliver at a facility and address pressing concerns around disclosure of HIV status. Agency and awareness of critical health issues and actions were augmented by frequent communication between the mobile-phone counselor and clients. Previous research has demonstrated that mobile-phone counseling is associated with improved outcomes for mother and child dyad (33, 34) and that virtual counseling is feasible and can be delivered via mobile phones in a community setting. In this study, mobile-phone counselors and women never met face to face, however, the narratives show that over time counselors and clients fostered a close relationship that conferred an additional layer of support for women. The close relationship built between the counselors and women was likely due to counselor attributes, focused counselor training, and anonymity of the counseling sessions that ensured that clients could express themselves freely without fear of being judged. This is an important finding, which is supported by other studies showing how mobile-phone counseling/messaging can provide anonymity and decrease levels of stress and feelings of isolation among new mothers, improve infant well-being and partner relationships, and facilitate adherence to ART (15, 20, 35, 36).

### Limitations

By design, qualitative studies may not be generalized to the larger population owing to the small sample sizes, however, they offer rich and in-depth nuances on participant experiences that can help inform the design of programs. In this study, qualitative interviews provided detailed insights into the key challenges, and facilitators and how mobile-phone counseling supported access to critical health services.

## Conclusions

Our findings from this qualitative study highlight the fundamental role mobile-phone counseling played in supporting HIV positive mothers enrolled in ANC by empowering them to address underlying individual, social, and structural factors associated with uptake of services. Study findings also highlight the need for future interventions to consider structural barriers to accessing health services (e.g., ensuring adequate nutrition for mothers to decrease the effects of poverty within communities and facilities). This study adds important insight to the small but growing body of research on the use of wireless and mobile technologies in health, more so in HIV and PMTCT care. The COVID-19 pandemic has also proven that mobile phones can be successfully used as a medium for doctor-patient consultations and relaying of COVID-19 test results.

## Data Availability Statement

The raw data supporting the conclusions of this article will be made available by the authors, without undue reservation.

## Ethics Statement

The studies involving human participants were reviewed and approved by Population Council's Institutional Review Board, the National Commission for Science, Technology, and Innovation (NACOSTI), and the Kenyatta National Hospital/University of Nairobi Ethics and Research Committee. The patients/participants provided their written informed consent to participate in this study.

## Author Contributions

JO: conceptualization, formal analysis, investigation, methodology, project administration, supervision, validation, writing—original draft, and writing—review and editing. AS: conceptualization, investigation, project administration, supervision, writing—original draft, and writing—review and editing. DL: formal analysis, validation, writing—original draft, and writing—review and editing. JM: formal analysis, supervision, and writing—review and editing. DO: supervision and review and editing. EK: project administration and review and editing. SK: project administration and writing—review and editing. All authors contributed to the article and approved the submitted version.

## Funding

This study was made possible through support provided by the US President's Emergency Plan for AIDS Relief and the US Agency for International Development (USAID) *via* HIVCore, a Task Order funded by USAID under the Project SEARCH indefinite-quantity contract (contract no. AID-OAA-TO-11-00060). The Task Order is led by the Population Council in partnership with the Elizabeth Glaser Pediatric AIDS Foundation, Palladium and the University of Washington.

## Conflict of Interest

JO, JM, AS, and SK were employed by Population Council. The remaining authors declare that the research was conducted in the absence of any commercial or financial relationships that could be construed as a potential conflict of interest.

## Publisher's Note

All claims expressed in this article are solely those of the authors and do not necessarily represent those of their affiliated organizations, or those of the publisher, the editors and the reviewers. Any product that may be evaluated in this article, or claim that may be made by its manufacturer, is not guaranteed or endorsed by the publisher.
